# Towards better Data Science to address racial bias and health equity

**DOI:** 10.1093/pnasnexus/pgac120

**Published:** 2022-07-26

**Authors:** Elaine O Nsoesie, Sandro Galea

**Affiliations:** Boston University School of Public Health, 715 Albany Street, Boston, MA 02118, USA; Center for Antiracist Research, Boston University, 120 Ashford Street, Boston, MA 02215; Boston University School of Public Health, 715 Albany Street, Boston, MA 02118, USA

## Abstract

Data Science can be used to address racial health inequities. However, a wealth of scholarship has shown that there are many ethical challenges with using Data Science to address social problems. To develop a Data Science focused on racial health equity, we need the data, methods, application, and communication approaches to be antiracist and focused on serving minoritized groups that have long-standing worse health indicators than majority groups. In this perspective, we propose eight tenets that could shape a Data Science for Racial Health Equity research framework.

Data Science is a multidisciplinary field that combines statistics, mathematics, and computer science with domain knowledge to extract and explain insights from data. The field of Data Science is driven by the ever-increasing generation of large volumes of varied data (including text, video, audio, and numbers) from digital devices, sensors, remote sensing and other technologies, and the opportunities to use these data to create change across different industries. The availability of these data hold the potential to better characterize how we live and as such have utility for population health science that underpins efforts to create a healthier world.

In the area of population health science, Data Science may have particular utility to address health inequities, wherein minoritized groups have long-standing worse health indicators than do majority groups. Traditional approaches to studying health inequities particularly racial inequities, have focused on identifying differences in health indicators without addressing the underlying processes, policies, and systems that create and propagate them. Data Science, in allowing us the capacity to more fully assess these conditions, may then hold the potential to improve health, and in particular to help narrow long intractable racial health gaps. Several authors have commented on the potential of Data Science to be transformative towards reducing health inequities (1–4). For example, by analyzing satellite and street view images of neighborhoods, we can develop comprehensive measures of the built environment that can characterize social determinants of health needs and point to ways in which improved access to built environment resources can improve health (5, 6). In another example, the use of mobile phone data during the COVID-19 pandemic allowed for a better understanding of the socioeconomic influences that shaped response to stay-at-home orders (7). Also, data mining was used to characterize gaps in COVID-19 racial data reporting in the United States (8).

Despite this potential, the contribution of Data Science to our efforts to narrow health inequities has been hampered by a field that is plagued by data and algorithms that are predicated on racist and White supremacist perspectives. We define White supremacist perspectives as the conscious or unconscious propagation through processes and policies of the belief that White people are superior and/or that the experiences of White people are the norm. For example, an algorithm used for parole and probation hearings—intended to reduce unintentional bias of the presiding judge—often predicted that Black defendants were at a higher risk of recidivism than they were, while White defendants were often classified as having a lower risk of recidivism than they were (9). In another example, an algorithm used in hospital management was less likely to refer sicker Black patients to receive additional resources compared to White patients (10). In both cases, the algorithms were trained on large volumes of data that included several demographic characteristics with the exclusion of race. However, policing and health insurance data are themselves shaped by the ubiquitous role of race in shaping daily lived life in America, meaning that the exclusion of race as a variable in these models did not remove racial bias.

There exists, at this point, a wealth of scholarship—including papers, books, essays, and (some) movies—discussing the ethical issues associated with the use of Data Science methods to address social problems. However, often lost in this conversation is the centrality of racial bias in Data Science and how we need to address this racial bias to the end of generating Data Science that can improve health equity. This race-conscious approach could help guard against the replication of problems that already exist by ensuring that the data, methods, and communication approaches are antiracist and focused on vulnerable communities.

The current shortcomings of Data Science suggest a need to develop a Data Science for Racial Health Equity research framework that focuses on how Data Science methods can be intentionally adapted and applied to improve racial health equity. Data Science for Racial Health Equity should be guided by principles that acknowledge and address racial and ethnic biases in data, methods, and applications. We provide relevant recommendations to advance the field toward a more racial and health equity focus.

## Guiding principles for a Data Science for Racial Health Equity

A Data Science for Racial Health Equity can be defined as a multidisciplinary field involving the application of mathematics, statistics, computer science [including machine learning and artificial intelligence (AI)], visualizations, storytelling, social science, and methods from domains not typically represented (such as law and ethics) to large volumes of data to (1) identify and quantify racial inequities; (2) link racial inequities to specific processes, systems, and policies; and (3) propose solutions that will eliminate or minimize these racial inequities.

A Data Science for Racial Health Equity can be guided by ethical and antiracist principles that are underpinned by the following eight tenets, some of which apply to the use of Data Science to address health equity more broadly.

1. *All datasets should be robustly interrogated*. Public health datasets are imperfect i.e. datasets embed personal, societal, and political biases. Data Science for Racial Health Equity should focus on racial biases in data. This would involve questioning every step of the Data Science process including data collection, processing, maintenance, analyses, and communication (11). Relevant questions include the following:
  - Who collected the data?
  - How was the data collected?
  - Who paid for the data collection?
  - What assumptions were made?
  - What populations are included/excluded?
  - What variables are included?
  - What are potential biases in the data?
  - How will the data be used?
  - How can algorithms trained with the data be misused?
2. *Human data are not a commodity*. Data are a representation of human experiences, biological and physiological processes. The frequently used references to data as a “commodity” or “the new oil” strip away the human elements in data (12). This is one of the reasons data automation in many industries is problematic because its focus is on optimization of processes, rather than the service of people, especially minoritized populations. Data Science for Racial Health Equity should aim to collect data that represent all relevant groups, especially marginalized populations. It should also disaggregate data by demographics and to the individual level (while protecting individual privacy) because aggregated data mask inequities. Furthermore, it should share data and tools with communities most affected by racial health inequities. At its core, the problem of data ownership is power. The sharing of data and findings combined with the capability to collect, manage, analyze, and use data effectively can shift power to marginalized communities by enabling these communities to use data to advocate for change.
3. *Humans are responsible for the biases perpetuated by algorithms*. We create the algorithms that we use in processing, analyzing, and communicating data. Each of us carry biases that we embed in the tools that we create. As products of a racist society, we are all capable of creating products that are racist. Data Science for Racial Health Equity needs to be antiracist, implying that data scientists should be actively seeking to understand and address bias in data and algorithms that promote racism and strive to change their internalized racism. In addition, Data Science for Racial Health Equity should intentionally develop algorithms that do not perpetuate racism, develop methods for assessing the potential racist impact of tools prior to deploying in the world, audit deployed algorithms regularly to assess actual impact, and carefully evaluate tools developed by others prior to using.
4. *Data should be studied through a historical lens*. A Data Science for Racial Health Equity needs to be founded on an understanding of the history of racism, recognizing that this is critical for social change. Data Scientists do not need to be historians but should understand the link between present day racial inequities and past policies. They should understand how frameworks such as Critical Race Theory and Settler Colonialism theories can improve the interpretation of racial data and how these frameworks and similar methods can be combined with Data Science methods to uncover and address racial inequalities.
5. *A Data Science for Racial Health Equity should be solution driven*. There are many decades of research showing health disparities across racial groups. It is certainly acceptable to identify problems even when we have not yet thought of appropriate solutions. However, to drive change, Data Science needs to focus both on identifying problems and solutions. For example, antiracist policies such as the eviction and mortgage relief moratoria that were implemented in the United States and other countries during COVID-19 can serve as a model for other antiracist policies that aim to mitigate the consequences of the centuries’ long disadvantage that racial minorities have faced in accumulating wealth (13, 14).
6. *A Data Science for Racial Health Equity should be collaborative*. Data Science broadly is not representative of populations (15–17). A Data Science for Racial Health Equity should be inherently collaborative by (i) including communities affected by racial inequity in conversations about project design and proposed solutions; (ii) creating opportunities to ensure that individuals in marginalized communities receive training and resources needed (including data and computing infrastructure) to be successful Data Scientists; and (iii) bringing together ideas from different fields particularly those not typically represented in Data Science, such as Law and History. Developing antiracist tools would require collaboration across disciplines (e.g. computer scientists working with public health researchers and policy scholars) and the development of new methods that combine ideas from different fields (e.g. a mixed-methods approach that combines AI and Critical Race Theory).
7. *Data need to be collected across multiple dimensions that often intersect*. Intersectionality theory explains how identities are shaped at the interstices of characteristics (e.g. gender and race) and as such it is critical to collect data that can be analyzed at these interstices (18). Big data also create the potential for disaggregation by several of these characteristics, in multiple combinations. Disaggregated data can reveal hidden inequalities and biases, and help us think critically about solutions, even ones that may not be a priori apparent at the time of data collection.
8. *A Data Science for Racial Health Equity should include the social determinants of health*. A Data Science for Racial Health Equity needs to capitalize on its potential to assess the context of health data and the many factors that affect how data are collected and shared, including political and monetary factors. A Data Science for Racial Health Equity needs to understand the biases embedded in data, at multiple levels of influence, in order to facilitate the evaluation of the impact of macro-level shifts on the health of populations. This suggests an opportunity to move beyond documenting inequities and identify sustainable solutions that rest on shifting the ubiquitous macro-social determinants of health.

In order for the approach proposed to be effective, we need to ensure its implementation in the education of data scientists, and that it informs both population health science research and the implementation of public health action. For example, a public health degree curriculum in Data Science should include courses on the history of racial injustice, on ethics, and on perspectives from Africa and other regions usually excluded. Similarly, population health science scholarship can move beyond considering race as a control variable and aim to understand the life circumstances that shape asset acquisition and that eventually produce health.

## Conclusion

Data Science can guide us towards narrowing health gaps through embracing large and diverse types of data that can better understand health inequities and propose solutions aimed at transforming systems and processes. We propose eight tenets that can guide a Data Science for Racial Health Equity. We suggest that incorporating these tenets into Data Science training, research, and implementation has the potential to guide us towards a more equitable world.
